# SEOM–SERAM–SEMNIM guidelines on the use of functional and molecular imaging techniques in advanced non-small-cell lung cancer

**DOI:** 10.1007/s12094-017-1795-y

**Published:** 2017-12-18

**Authors:** G. Fernández-Pérez, R. Sánchez-Escribano, A. M. García-Vicente, A. Luna-Alcalá, J. Ceballos-Viro, R. C. Delgado-Bolton, J. C. Vilanova-Busquets, P. Sánchez-Rovira, M. P. Fierro-Alanis, R. García-Figueiras, J. E. Alés-Martínez

**Affiliations:** 1 Department of Radiology, Hospital Universitario Rio Hortega, Valladolid, Spain; 2 Department of Medical Oncology, Hospital Universitario de Burgos, Burgos, Spain; 3 Nuclear Medicine Department, University General Hospital, Ciudad Real, Spain; 4 Clínica Las Nieves, Health Time, Jaén, Spain; 5 Department of Radiology, University Hospitals of Cleveland, Case Western Reserve University, Cleveland, OH USA; 6 Oncology Unit, Oncología Médica, Hospital Nuestra Señora de Sonsoles, Complejo Asistencial de Ávila, C/Avda, Juan Carlos I, s/n, 05004 Ávila, Spain; 7 Department of Diagnostic Imaging (Radiology) and Nuclear Medicine, San Pedro Hospital and Centre for Biomedical Research of La Rioja (CIBIR), University of La Rioja, Logroño, Spain; 8 Servicio de Radiología, Instituto Catalán de Salud (IDI) Girona, Clinica Girona, Girona, Spain; 9 Department of Medical Oncology, Hospital Universitario de Jaén, Jaén, Spain; 10 Nuclear Medicine Department, Complexo Hospitalario Universitario de Santiago de Compostela, Santiago de Compostela, Spain; 11 Department of Radiology, Complexo Hospitalario Santiago de Compostela, Santiago de Compostela, Spain

**Keywords:** Non-small-cell lung cancer, Functional imaging, Multi-detector computed tomography, 18F-fluorodeoxyglucose positron emission tomography, Magnetic resonance imaging

## Abstract

Imaging in oncology is an essential tool for patient management but its potential is being profoundly underutilized. Each of the techniques used in the diagnostic process also conveys functional information that can be relevant in treatment decision-making. New imaging algorithms and techniques enhance our knowledge about the phenotype of the tumor and its potential response to different therapies. Functional imaging can be defined as the one that provides information beyond the purely morphological data, and include all the techniques that make it possible to measure specific physiological functions of the tumor, whereas molecular imaging would include techniques that allow us to measure metabolic changes. Functional and molecular techniques included in this document are based on multi-detector computed tomography (CT), 18F-fluorodeoxyglucose positron emission tomography (18F-FDG PET), magnetic resonance imaging (MRI), and hybrid equipments, integrating PET with CT (PET/CT) or MRI (PET-MRI). Lung cancer is one of the most frequent and deadly tumors although survival is increasing thanks to advances in diagnostic methods and new treatments. This increased survival poises challenges in terms of proper follow-up and definitions of response and progression, as exemplified by immune therapy-related pseudoprogression. In this consensus document, the use of functional and molecular imaging techniques will be addressed to exploit their current potential and explore future applications in the diagnosis, evaluation of response and detection of recurrence of advanced NSCLC.

## Introduction

Imaging in oncology is more than a purely morphological description. Each of the techniques used in the diagnostic process is also adding functional information that can be relevant in treatment decision-making. However, this information, which is often implicit in the acquired data, is not generally used or is done so only superficially. Furthermore, new imaging algorithms and techniques are currently being developed that enhance our knowledge about the phenotype of the tumor and its potential response to different therapies. It is an emerging field that calls for the close collaboration of each professional involved to understand the interaction between tumor biology and image, with the aim of incorporating these advances into clinical practice in a useful manner. Consequently, functional imaging can be defined as the type of imaging that provides information beyond the purely morphological data, informing about the nature of the tumor in its baseline status and the changes induced by the effect of various therapeutic interventions.

Functional imaging would, therefore, include all techniques that make it possible to measure specific physiological functions of the tumor, such as perfusion, or those that have relation with water molecule mobility (diffusion), whereas molecular imaging would include techniques that allow us to measure metabolic changes depending on the macromolecules or tracers used [1]. Functional and molecular techniques included in this document are based on multi-detector computed tomography (CT), 18F-fluorodeoxyglucose positron emission tomography (^18^F-FDG PET), magnetic resonance imaging (MRI), and hybrid equipment, integrating PET with CT (PET/CT) or MRI (PET-MRI).

Lung cancer is one of the most frequent and deadly tumors although survival is increasing thanks to advances in diagnostic methods and new treatments. For instance, mediastinal staging with the incorporation of minimally invasive techniques, such as endobronchial ultrasound (EBUS), and non-invasive techniques, such as PET/CT, allows for the better selection of patients that can benefit from potentially curative surgical or radiation therapy treatments. The pathological characterization is evolving even more rapidly. From a simple dichotomous model (small-cell lung cancer, SCLC, *versus* non-small-cell lung cancer, NSCLC) it went to another one, in which different treatment strategies were associated to different pathological entities within NSCLC, such as squamous carcinoma and adenocarcinoma. And from there to a complex landscape, in which different driver mutations, translocations, and immunology markers are needed to define the more proper course of action for any given patient. Thus, different molecular subtypes are susceptible to specific targeted treatments, such as those aimed at the epidermal growth factor receptor (EGFR) [2] or at the translocation of the anaplastic lymphoma kinase (ALK) gene [3], greatly impacting on patients’ survival and quality of life in advanced stages of the disease, as have the introduction of immunotherapy as a treatment for lung cancer [4, 5]. The increased survival also poises challenges in the terms of proper follow-up and definitions of response and progression, as exemplified by immune-related pseudoprogression [6].

In this consensus document, the use of functional and molecular techniques will be addressed in advanced NSCLC with the following objectives:

1. To define the applications of functional and molecular imaging techniques in the diagnosis of advanced NSCLC.
  1.1. Correlation with histopathology and prognostic capacity.
  1.2. Detection of tumor spread (staging).
    1.2.1. Extension of the primary tumor (T).
    1.2.2. Regional lymph node spread (N).
    1.2.3. Distant disease (M).
2. Applicability of functional and molecular imaging techniques in evaluating response to conventional treatments, targeted and antiangiogenic therapies, and immunotherapy.
  2.1 Evaluation of response and use in follow-up
    2.1.1. Morphological CT.
    2.1.2. Functional tests that evaluate metabolic information (^18^F-FDG PET).
    2.1.3. Functional tests that assess tumor perfusion (dynamic studies with contrast).
    2.1.4. Functional tests based on tumor microstructure (diffusion-weighted imaging, DWI).
  2.2. Challenges in evaluating response
3. Future perspectives for imaging techniques

## To define the applications of functional and molecular imaging techniques in the diagnosis of advanced NSCLC

### Correlation with histopathology and prognostic capacity

**Fig. 1:**
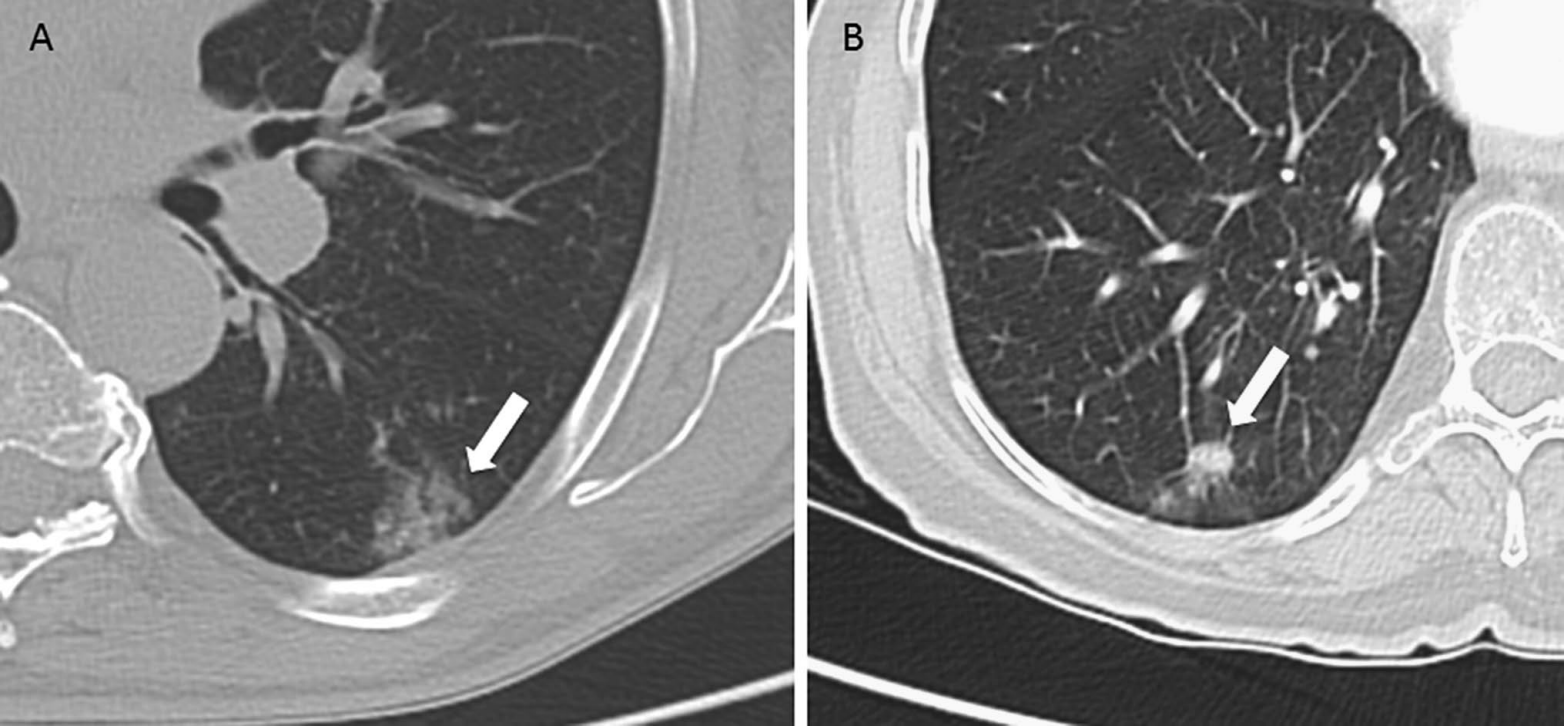
Role of thoracic CT in the characterization of pulmonary nodules. **a** 75 year-old male with a 2.5 cm subsolid nodule that experienced no change during 2 years of follow-up CT (arrow). The biopsy showed in situ adenocarcinoma. **b** Patient with a subsolid nodule that includes a solid component (arrow). Pathology exam showed an invasive adenocarcinoma

Structural imaging techniques supply information useful for the initial or tentative histopathological diagnosis, particularly with regard to the subtypes of adenocarcinoma and their potential risk of malignancy. In CT, the term, “subsolid nodule” refers to the “ground glass” component of a nodule. The International Association for the Study of Lung Cancer (IASLC), American Thoracic Society (ATS), and the European Respiratory Society (ERS) consider that when a persistent nodule is solely subsolid and measures less than 5 mm, it is a focus of atypical adenomatous hyperplasia, and when it is larger, but less than 3 cm, the possibility of in situ adenocarcinoma or minimally invasive adenocarcinoma should be considered. Larger size and, especially in association with a solid component, proportionally increases the potential of malignancy [7, 8]. (Fig. 1).

The functional and molecular information obtained from imaging techniques is useful in differentiating between benign and malignant pulmonary nodules, despite the limitations of these techniques in characterizing lesions measuring < 5 mm. Thus, perfusion-CT, ^18^F-FDG PET/CT, diffusion-weighted imaging (DWI), and perfusion-MRI techniques have proven their reliability above 90% for this differentiation [9]. A negative, linear correlation has also been shown between DWI-derived quantitative biomarkers and NSCLC, particularly with the different subtypes of adenocarcinoma [10]. Furthermore, the combined use of DWI and PET (measuring the maximum standardized uptake value or SUV_max_) results in a more useful stratification of patients with adenocarcinoma in different prognostic subgroups compared to the independent use of each technique [11].

### Detection of tumor spread (staging)

Staging of lung cancer includes detection and characterization of the primary tumor and an in-depth study of the local, regional, and distant dissemination to orientate the most appropriate therapy. Imaging plays an essential role in the clinical or cTNM classification, with CT being the method of choice, given its wide availability [12]. In addition, ^18^F-FDG PET/CT has become another standard tool in pre-treatment assessment, with advantages over conventional techniques, for staging both the mediastinum [13, 14], as well as extrathoracic disease [15] having demonstrated it lowers the number of useless thoracotomies, and improves cost-effectiveness in the management of these patients [16]. Therefore, given the importance of an integrated morphological-functional assessment for proper treatment planning, one technique without the other is not understandable. In the latest revision of the National Comprehensive Cancer Network (NCCN), when contemplating surgical treatment, the recommendation is that CT and PET/CT should be performed within a period of time no greater than 60 days before surgery [17].

When attempting to discern lung cancer resectability, CT uses Glazer et al.’s three radiological criteria for mediastinal infiltration [18]. Contact with the mediastinum measuring less than 3 cm, loss of integrity of a fatty plane, and a circumferential contact of less than 90º with the thoracic aorta. These criteria can be correctly applied in most situations. However, a non-resectable tumor is more difficult to define; thus, in a series of 48 surgically resectable lung cancers, 21 of them presented more than 3 cm of contact with the mediastinum [19, 20]. Subsequent studies demonstrated that the criteria of contact with the aorta and loss of a fatty plane exhibited a sensitivity of 40 and 27%, respectively [19, 20]. These results are not surprising, since patient movement or partial volume artifacts are not uncommon on CT. Nevertheless, the technological improvements of CT in recent years, both in temporal as well as spatial resolution (submillimetric acquisitions), even with the capacity to perform studies with cardiac synchronization, has enhanced it diagnostic capacity, reaching 86% sensitivity, 96% specificity, and 95% accuracy [21]. The complementary use of perfusion-CT or dual energy exams increases this diagnostic capacity, elevating the possibilities that radical or potentially curative treatments will be of benefit [22].

#### Extension of the primary tumor (T)

In characterizing the primary tumor, PET improves the delimitation of central tumors associated with atelectasis or post-obstructive pneumonitis, a critical feature in planning radiotherapy [23, 24]. Moreover, according to a recent meta-analysis, PET can aid in defining tumor spread to the pleura with overall sensitivity and specificity of 86 and 80%, respectively [25].

**Fig. 2:**
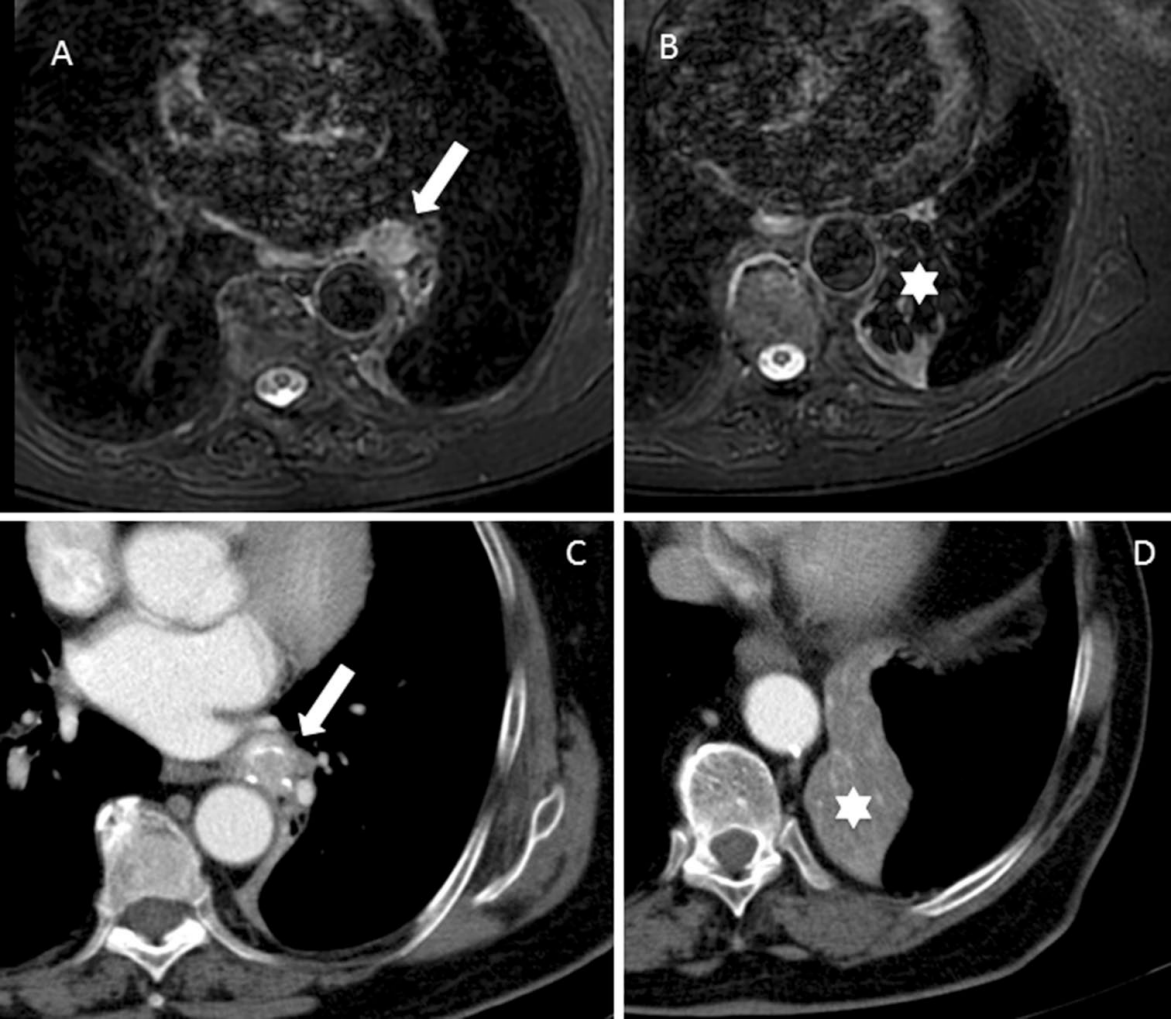
Comparison between STIR sequence and CT for differentiation between atelectasis and NSCLC. **a**, **b** STIR sequences where the tumor (arrow) and the associated atelectasis (asterisk) can be clearly identified and differentiated. However, in **c**, **d** CT images, the distinction between atelectasis and tumor is less evident

At present, morphological MRI has a recognized role to play in studying situations of possible spread to neural structures, as in tumors of the sulcus, where a very precise characterization is required to plan surgery or radiotherapy. The technological development of MRI is overcoming the initial difficulties in its application in the lung, by incorporating techniques with respiratory and cardiac synchronization that can provide greater sensitivity in ruling out mediastinal, hilar, or thoracic wall infiltration (Fig. 2). MRI can incorporate a variety of functional information to structural protocols through certain sequences, such as perfusion-MRI or DWI [26]. For instance, perfusion-MRI explores tumor angiogenesis and enhances differentiation between the subtypes of NSCLC, and is of great use in monitoring antiangiogenic drug treatment. On the other hand, DWI is a powerful indirect biomarker on tumor cellularity, by studying the Brownian movement of free water. Proven applications of this technique in NSCLC are a better differentiation between the tumor and associated atelectasis, distinction between different tumor subtypes, and aiding in assessing and predicting tumor response. This technique can be incorporated into “whole body” protocols in the staging of NSCLC with results that are similar to those of PET [27], something to be taken into account when there is difficult access to PET. Perhaps, the PET-MRI technique integrating whole body MRI and PET will add a new perspective in this area [28, 29].

#### Regional lymph node spread (N)

CT can identify even small lymph nodes. Size is a fundamental criterion. A lymph node diameter in the short axis greater than 10 mm will be considered pathological. However, this definition based solely on size has its limitations, since 37% of lymph nodes measuring 2–4 cm will not be metastatic and, in contrast, normal-sized nodes can harbor secondary tumor infiltration, in particular if the primary tumor is an adenocarcinoma [30, 31]. The quantification of contrast attenuation in Hounsfield units can improve these results, principally when the nodes display a round morphology or when there is peripheral enhancement and central necrosis. In a recent study conducted in 37 patients, a good correlation was seen between attenuation measurements in lymph nodes on CT and SUV values on ^18^F-FDG PET [32].

PET/CT is an indispensable tool in the assessment of mediastinal lymph nodes. In a recent meta-analysis, the overall sensitivity and specificity values were 62 and 92%, respectively [33]. The differences in sensitivity and specificity results among series are due to the different prevalence of infectious pathology and tumor histological variety, with more limited positive and negative predictive values for adenocarcinoma with respect to squamous cell carcinoma (50 and 78 vs. 23 and 96%, respectively) [34]. In spite of its high specificity, the rate of false positives can decrease treatment options, especially in patients living in endemic areas for granulomatous disease [35]. Therefore, nodal extension study should also integrate morphological and attenuation criteria (e.g., presence of calcifications). In addition, ^18^F-FDG PET presents limitations in nodal staging of centrally located primary tumors [36, 37].

On the other hand, in early stages, given the high negative predictive value of ^18^F-FDG PET’s in areas where granulomatous disease is not endemic (sensitivity close to 100%), it could prevent the use of invasive or minimally invasive techniques, and the same would happen with enlarged lymph nodes that are negative on ^18^F-FDG PET. Even so, there is not sufficient evidence to preclude histological confirmation of ^18^F-FDG PET/CT findings. In addition, when performing ^18^F-FDG PET/CT studies, special attention must be paid regarding the chosen protocol and the standardization of the procedure, to obtain reproducible results [38].

Several studies have reported on the usefulness of MRI in identifying mediastinal lymph node involvement, notably using the so-called short time inversion recovery (STIR) sequences, where mediastinal lymph nodes exhibit a higher signal than non-suspicious nodes, with sensitivity and specificity values of 90 and 92%, respectively [39]. However, the possible advantage of this technique over ^18^F-FDG PET/CT remains to be defined [40].

#### Distant disease (M)

The presence of distant metastases is common at the time of NSCLC diagnosis and a correct detection is crucial to avoid unnecessary surgery and select the appropriate systemic therapy. Lung cancer metastases are usually hypovascular on CT, although in other organs, such as the brain, they can be hypervascular or highly dense on the baseline study, indicating bleeding or hemorrhage inside the tumor. On the other hand, there can be metastatic disease in which local management is of special importance, as in cases of single thoracic or extrathoracic metastases (stages M1a and M1b of 8th ed. of the TNM). For example, in the case of brain metastases, performing an MRI is critical to establish the most appropriate type treatment, specifically stereotactic radiotherapy [40].

^18^F-FDG PET/CT significantly raises the rate of detection of extrathoracic metastatic disease up to 15% more than with conventional methods, like CT or bone scintigraphy, with 93% sensitivity and 96% specificity. Its greatest limitation lies in the detection of cranial metastases due to brain’s high physiological uptake of ^18^F-FDG. In multicenter studies, ^18^F-FDG PET/CT has shown its superiority in the detection of extracranial metastases, preventing up to 14% of unnecessary surgeries and proving that it is the best imaging method for staging NSCLC in the early stages of the disease [41–44].

**Fig. 3:**
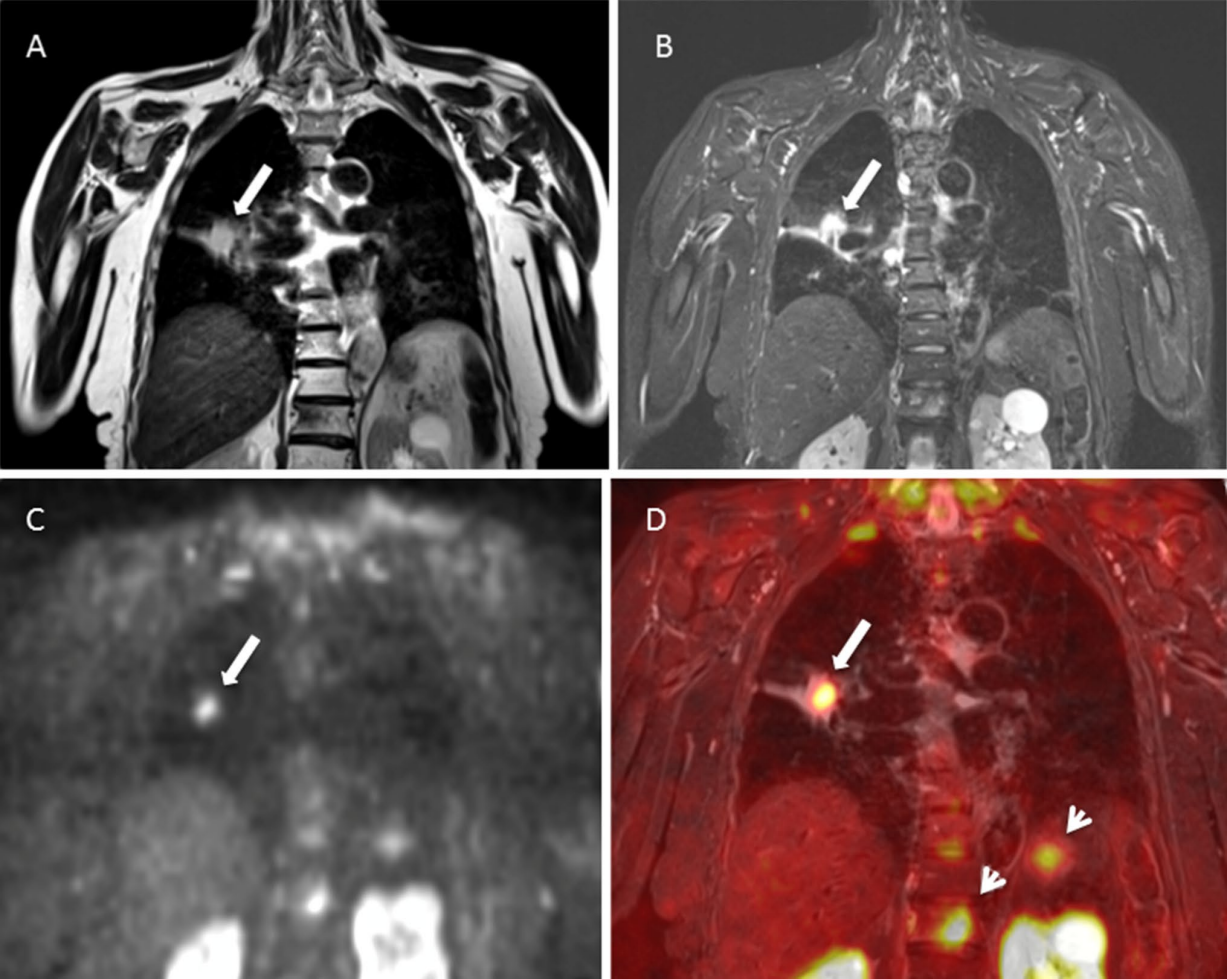
MRI in patient with lung adenocarcinoma. **a** T2-weighted and **b** STIR sequences. A nodule in the right lung parenchyma can be identified, which is contact and also thickens the minor fissure with associated atelectasis (arrow). **c** High b-value (1000 s/mm^2^) DWI shows increased signal of the pulmonary nodule (arrow), without high signal in the minor fissure consistent with a malignant origin and lack of fissure involvement. **d** Fused image of coronal T2-weighted image and DWI better depicts the nodule (arrow) and metastatic lesions at L1 vertebral body and left adrenal gland (arrowheads) with advantage over morphological sequences (**a**, **b**)

MRI is especially valuable in detecting and evaluating disease in the brain, particularly in the context of single metastasis or oligometastatic disease. In relation to other locations, MRI has been reported to be advantageous in the detection of bone and liver lesions (Fig. 3), and PET is more apt for the involvement of lymph nodes and soft tissue [44–46]. The development of the “moving table” technology and the use of multiple surface phase-array coils makes it possible to perform whole-body scans, with morphological and functional sequences with significantly shortened examination times [47, 48].

## **A**pplicability of functional and molecular imaging techniques in evaluating response to conventional treatments, targeted and antiangiogenic therapies, and immunotherapy

### Evaluation of response and use in follow-up

#### Morphological CT

The response to treatment is nowadays evaluated by CT on the basis of the strictly standardized morphological criteria of the RECIST system (Response Evaluation Criteria In Solid Tumors) v1.1. Nevertheless, there are situations, especially following treatment with the new modalities of immunotherapy and targeted molecular drugs, in which tumors that respond to treatment can be erroneously categorized as progression or stable disease. Different studies have demonstrated that the reduction of size assessed by CT is not always a good predictor of patient survival following treatment with EGFR tyrosine-kinase inhibitors (TKI), such as erlotinib, gefitinib, or afatinib, mainly because lesions may grow initially, representing the so called “pseudoprogression” [49].

#### Functional tests that evaluate metabolic information (^18^F-FDG PET)

Despite the availability and good concordance of the European Organisation for Research and Treatment of Cancer (EORTC) and Positron Emission tomography Response Criteria In Solid Tumors (PERCIST) criteria to evaluate metabolic response using ^18^F-FDG PET/CT, its use is still not widespread [50–52]. This lack of standardization in the determination of metabolic response limits the reproducibility of the results and the consideration of ^18^F-FDG PET/CT as a recommended technique to evaluate response in current oncological management guidelines [17].

During follow-up, distinguishing tumor persistence/recurrence from postoperative/post-radiotherapy changes continues to be a true challenge for both CT and PET. Nevertheless, on ^18^F-FDG PET, a diffuse increase in uptake that gradually decreases over time usually indicates that this finding is benign, whereas if it is focal or increases over time, it should be complemented when feasible with a confirmatory biopsy to rule out tumor viability [53, 54]. The same technical procedure should be applied in the baseline and in the follow-up when evaluating response to therapy with ^18^F-FDG PET [38].

Choi et al. followed up 358 patients with resected NSCLC using CT and ^18^F-FDG PET/CT and found that recurrence was only detected with ^18^F-FDG PET/CT in 37% of the cases, although it failed to detect 6 small hypometabolic lesions. Therefore, they suggested including an annual PET/CT in the follow-up algorithm [55]. Following chemo- or radiation therapy, it is recommended to wait for at least 3 months before performing a follow-up ^18^F-FDG PET/CT, to avoid metabolic uptake secondary to post-radiation pneumonitis. Areas treated with radiotherapy may remain avid for ^18^F-FDG for even 2 years after treatment. However, given its high negative predictive value, a negative PET/CT would rule out relapse [56]. However, current guidelines specifically recommend against the routine use of PET/CT in NSCLC follow-up. This reasoning may change in the future with the advent of more efficacious treatments in the metastatic and/or relapse setting [17].

According to the diagnostic performance of ^18^F-FDG PET/CT in tumor restaging after induction treatment with chemotherapy and/or radiotherapy, a systematic review concludes that the rate of false negatives for both CT and PET is a limiting factor in the detection of pulmonary disease. Regarding mediastinal node restaging, the rate of false negatives and false positives is too high both for ^18^F-FDG PET and CT, although it is slightly lower for ^18^F-FDG PET than for CT (25 and 33%, for ^18^F-FDG PET, respectively vs. 40 and 40%, for CT, respectively). Therefore, neither of these techniques is a good marker of residual disease and, consequently, they are not suitable for guiding surgical planning [57].

In contrast, a retrospective study of 545 patients who underwent ^18^F-FDG PET during induction treatment demonstrated an association between increased tumor metabolism and worse prognosis. Moreover, in this study, a change in the metabolism of mediastinal lymph nodes was the most robust predictor of survival in the multivariate analysis [58].

Consequently, in practical terms, the greatest advantage of ^18^F-FDG PET/CT in the evaluation of response and follow-up of NSCLC would lie in the determination of local and distant progression.

**Fig. 4:**
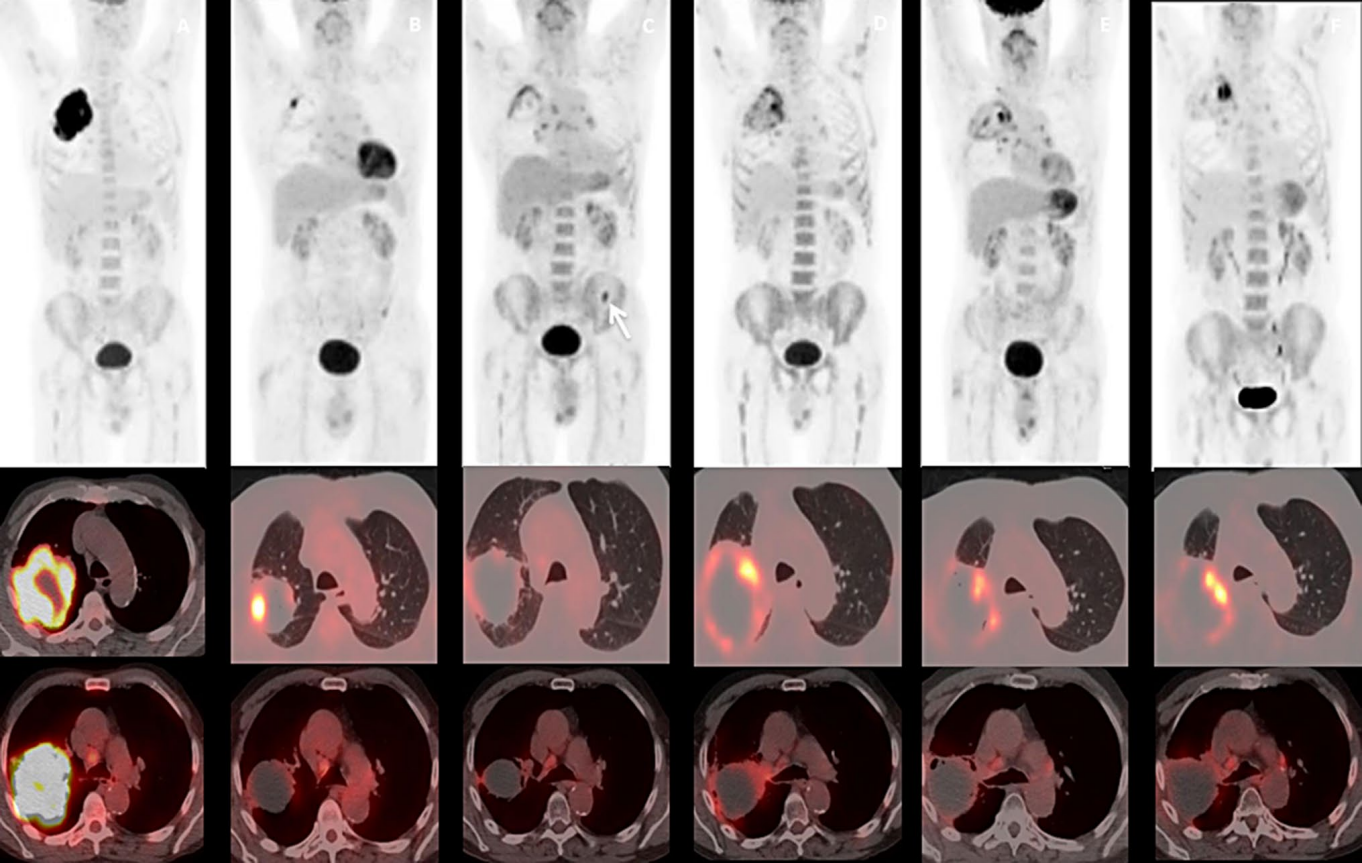
73 year- old male patient diagnosed with NSCLC (wild type EGFR and ALK adenocarcinoma). T3N2 clinical stage. **a** Basal ^18^F-FDG PET/CT shows hypermetabolic right lung mass with ipsilateral mediastinal involvement. **b** PET/CT after chemotherapy (Ch) shows partial response. Patient underwent radiotherapy (RT) and second line of Ch. C. PET/CT 3 months after RT shows metabolism in lung mass, probably related with persistence of disease although concomitant inflammation could not be discarded. Hypermetabolic uptake in multiple mediastinal and hilar lymph nodes, probably secondary to inflammation. FDG uptake in left hemipelvis in correspondence with descendent colon (arrow). Colonoscopy demonstrated an adenoma with moderate dysplasia. **d** Follow-up PET/CT demonstrated progression. Immunotherapy was initiated. **e**, **f** PET/CTs during immunotherapy treatment show disease stability

Personalized treatment is still a challenge and requires monitoring the biological parameters responsible for treatment resistance. ^18^F-FDG PET is a well-established technique that can quantify biological parameters that are important for both local and systemic therapy. In this sense, a review published in 2014 analyzed the assessment of response using ^18^F-FDG-PET/CT in patients with NSCLC treated with EGFR TKI. Seven studies summing up 210 patients were included and an association was demonstrated between metabolic response and clinical and radiological response, as well as survival. Even further, when there was a significant reduction of metabolic activity 1–2 weeks after initiating an anti-EGFR treatment, maintaining this therapy was seen to be beneficial for the patient. In contrast, lack of an early response to EGFR TKI indicated treatment inefficacy [59]. In addition, another recent study in ALK-positive NSCLC examined metabolic response in comparison with CT following treatment with crizotinib, ALK inhibitor that induces a high response rate in this type of patients. A baseline ^18^F-FDG-PET/CT and another one after 6 weeks of treatment were performed in 15 patients. The response on CT was analyzed according to RECIST 1.1, whereas the response on ^18^F-FDG-PET/CT was analyzed using the EORTC 1999 and PERCIST criteria. The authors found that ^18^F-FDG-PET/CT detected disease progression before CT in almost half of the patients [60] (Fig. 4).

#### Functional tests that assess tumor perfusion (dynamic studies with contrast)

**Fig. 5:**
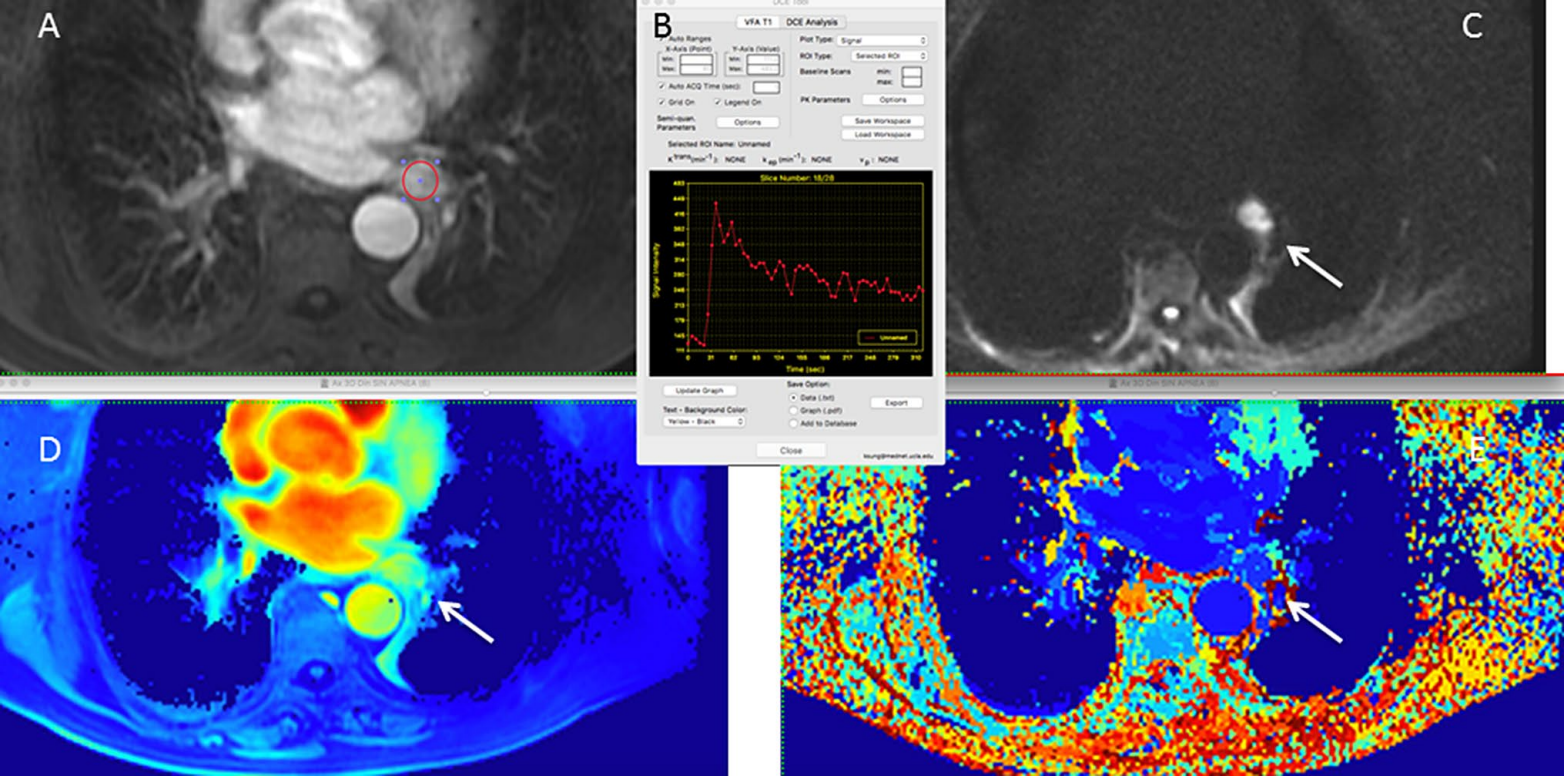
Evaluation of angiogenesis of NSCLC with perfusion MR (same patient of Fig. 2). Dynamic contrast-enhanced MR sequences. **a** Dynamic-enhanced postcontrast image shows a left hilar mass (ROI), which demonstrated in (**b**) the signal-intensity-time curve a fast uptake of contrast and delayed wash-out suggesting is malignant origin, as confirmed in (**c**) high b-value DWI where the lesions shows high signal intensity (arrow). **d**, **e** Semiquantitative perfusion maps of peak time and area under the curve better depicts the vascular characteristics of the tumor (arrows)

Dynamic-enhanced MRI and CT, also known as perfusion-MRI and perfusion-CT, respectively, can give information about neo-angiogenesis, contributing physiological data about blood flow, vascular volume, and tumor permeability. Initially, these techniques were used to characterize pulmonary nodules as benign or malignant, but now they are being applied in the assessment of treatment response. They use quantitative parameters, derived from a pharmacokinetic analysis, such as *K* ^trans^, an indicator of flow and tumor permeability, and *K*_ep_, which provides information about contrast diffusion toward the intravascular space. In clinical practice, semi-quantitative measurements and curves based on contrast enhancement during image acquisition are more often used (Fig. 5).

Perfusion-MRI is more complex to acquire because of breathing movements, making it necessary to use high temporal resolution acquisition strategies that are largely unsusceptible to movement and also to apply co-registration techniques during post-processing. Despite the limited clinical use of this technique, it has yielded excellent results in various clinical scenarios, such as pulmonary nodule characterization according to the morphology of the signal intensity-time curve, where it can be an alternative in cases in which neither the CT or ^18^F-FDG PET/CT have been conclusive [10].

Another scenario in which perfusion-MRI has aroused great interest is in the early evaluation of NSCLC treated with antiangiogenic drugs. However, variations in measurements and the use of different mathematical models to calculate these quantitative parameters limit reproducibility and comparison of results; hence, multicenter studies are needed to standardize them.

On perfusion-CT, a similar approach to perfusion MRI is used based on the injection of iodinated contrast and rapid acquisition to assess the contrast’s transit time within the tumor and normal tissues and its washout, thereby increasing the radiation dose in comparison to conventional CT acquisitions, which is an important limitation to its current inclusion in clinical protocols. Similar to perfusion-MRI, they can predict early treatment responses before the tumor begins to shrink. Several studies have yielded the decrease of vascular flow (BF) and permeability (PMB) values in tumors undergoing antiangiogenic treatment [61]. Nevertheless, higher uniformity is needed in how these studies are conducted with relation to the volumes and rate of contrast injection, dose technique, and quantification models [61, 62].

#### Functional tests based on tumor microstructure (diffusion-weighted imaging, DWI)

**Fig. 6:**
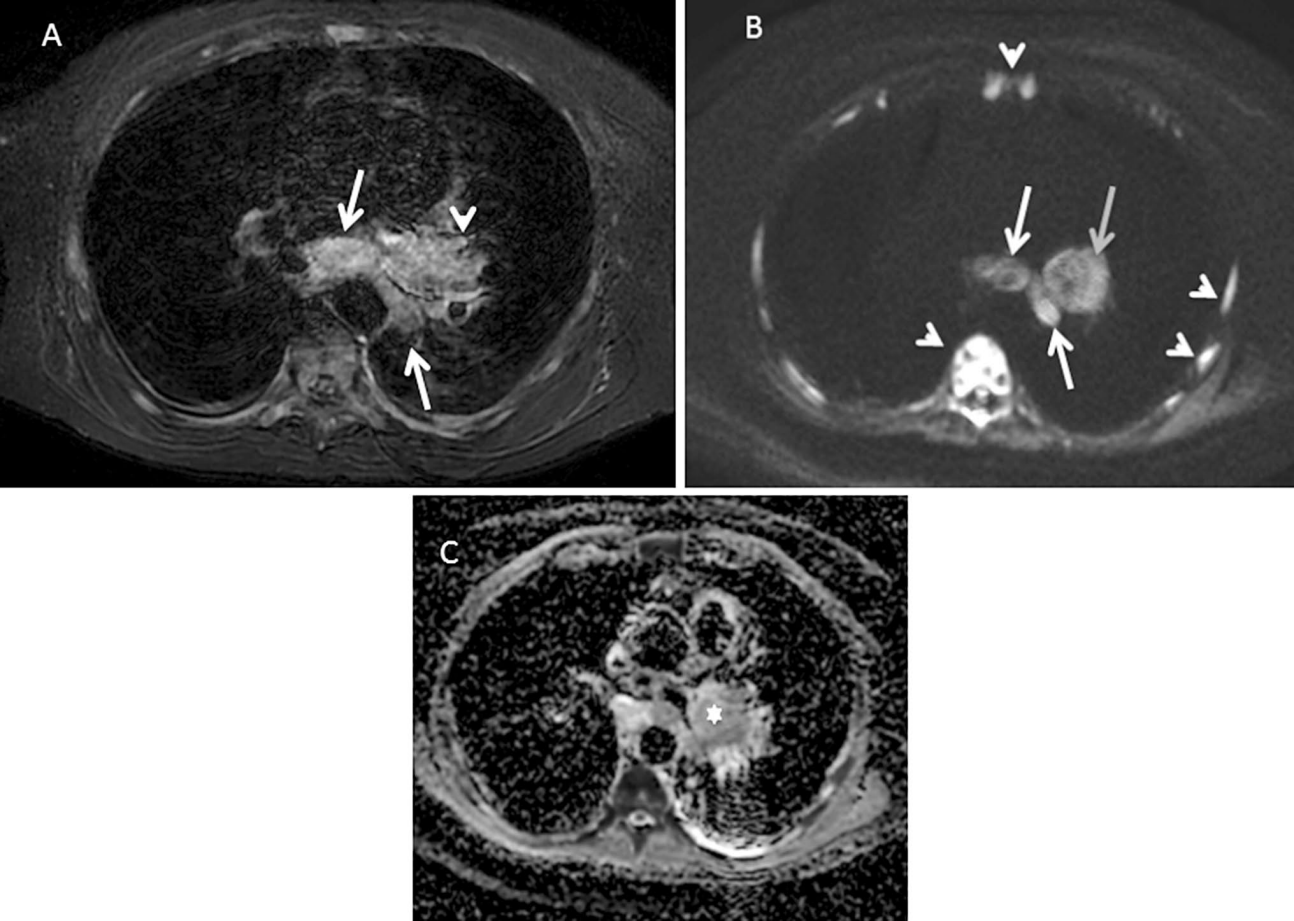
Locoregional MRI staging of NSCLC. **a** STIR shows a left hiliar mass in relation to adenocarcinoma (arrowhead) and also metastatic subcarinal and paraaortic adenopathies (arrowheads), **b** High b-value DWI shows a high signal intensity of both the primary tumor (gray arrow) and enlarged lymph nodes (white arrows), as well as in the thoracic vertebral body, left ribs and sternum (arrowheads) consistent with metastatic bone disease. **c** Low ADC value (0.8 × 10^−3^ mm^2^/s) of the left hilar mass (asterisk)confirms the high aggressiveness of the primary tumor

**Fig. 7:**
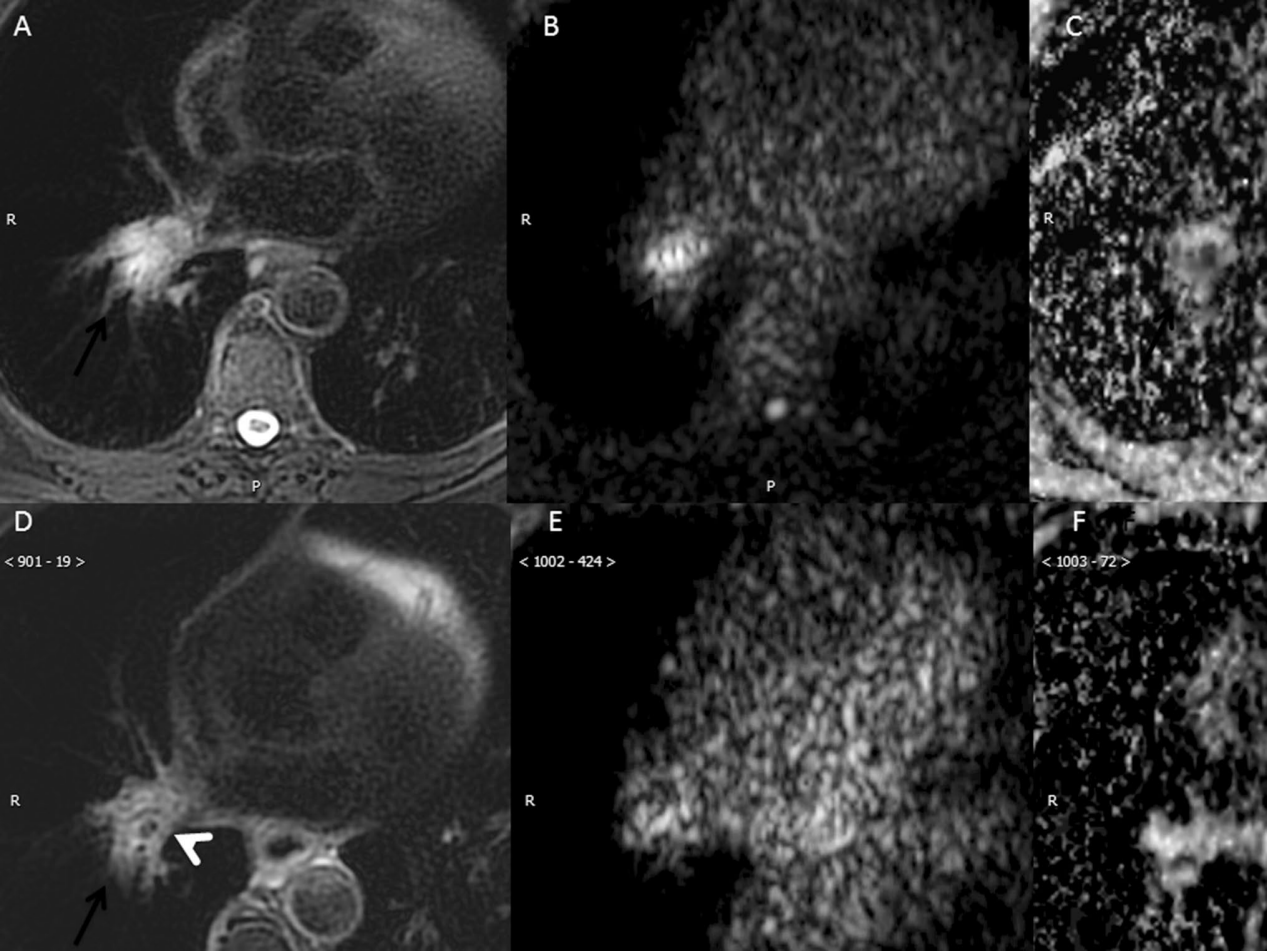
Follow-up MRI studies in a 62 year-old male with lung invasive adenocarcinoma stage IIIA (arrows) treated (**a**–**c**) Pretreatment MRI (top row images) shows a primary right hilar mass of 29 mm invading main and intermediate bronchi, which shows high signal intensity on fat-suppressed T2-weighted image (**a**) and high signal on high b value DWI (**b**) and low signal on ADC map (ADC: 1.18 × 10^−3^ mm^2/^s) (**c**), representing true restriction diffusion and related to the high aggressiveness of the mass. After 2 months of treatment neoadjuvant chemotherapy, a new MRI was performed to evaluate surgical options (bottom row images), demonstrating a significant reduction of the right hilar mass (maximum diameter of 19 mm) and with recanalization of the intermediate bronchus on fat-suppressed T2-weighted image (**d**). In addition, there is a reduction of the signal intensity of the lesion on high b value image (**e**) and increase of ADC value (1.58 × 10^−3^ mm^2^/s) representing good response to treatment. This case shows how DWI can help in treatment selection as a pretreatment low ADC value has been related to good response to chemotherapy

DWI is an MRI technique that provides information about the tumor microenvironment and can distinguish between areas of inflammation or fibrosis from areas with active tumor [63]. This technique evaluates the Brownian movement of water molecules in the tissue submitted to certain radiofrequency pulses [64]. Tumors have greater cell content, as well as membranes and a more complex cellular microenvironment that impedes the free movement of water molecules in the interstitial space (Fig. 6). This technique quantifies the restriction of diffusion of free water molecules using the apparent diffusion coefficient (ADC) that has been proposed as a surrogate marker of tumor aggressiveness and providing useful information in the early assessment of tumor response by the detection of subtle changes in tumor microstructure with treatment. Increased ADC following chemotherapy or radiotherapy for advanced NSCLC has been correlated with good response, as well as greater disease-free survival and overall survival [65, 66]. Preliminary data assign greater effectiveness to ADC than perfusion-MRI or ^18^F-FDG PET/CT parameters in treatment monitoring in NSCLC and point to a role in predicting response to chemotherapy, since a low pretreatment ADC value predicts a good response better than ^18^F-FDG PET [67–69] (Fig. 7). These early biomarkers of response would improve not only the cost-effectiveness of treatments in clinical practice, but might also contribute to improving the design of clinical trials for new drugs or treatment strategies.

In evaluating the phenomena of pseudoprogression associated with inflammation and tissue fibrosis, DWI has the possibility of mitigating the confounding effects of these growing non-tumor areas [70].

There are other promising MRI techniques, such as blood oxygenation level dependent (BOLD) sequences that make use of quantification of the T2* values of tumor and surrounding tissues by evaluating the inhomogeneity of the magnetic field associated with differences in the concentration of paramagnetic substances, like deoxyhemoglobin and that are potentially useful in determining tumor hypoxia after establishing a treatment [71].

### Challenges in evaluating response

The incorporation of other treatment strategies into the standard surgery-radiotherapy-chemotherapy triad in lung cancer, such as antiangiogenic drugs, targeted therapies for actionable mutations and immunotherapy with immune-regulating checkpoint inhibitors (anti-CTLA-4, anti-PD-1, and anti-PD-L1), has modified response patterns making them not entirely evaluable with classical morphological imaging criteria, such as RECIST, or even with molecular techniques, such as ^18^F-FDG PET. Thus, tumors that are responding to treatment could be mistakenly classified as in progression (pseudo-progression), with ensuing withdrawal of drugs that are actually active. It is critical that these situations can be properly interpreted, and the support of functional imaging methods can be decisive in this regard.

The assessment of early antiangiogenic response has been studied with perfusion-MRI, using quantitative parameters such as *K* ^trans^ and *K* ^ep^, with promising results [72]. In the case of patients with EGFR-mutated tumors treated with TKI, ^18^F-FDG PET has proven to be superior to CT in evaluating response [73]. Additional issues are posed with EGFR- and ALK-inhibitors. In this regard, continuing treatment even when the morphological criteria of progression (RECIST-PD) are met has been proven to be beneficial; in fact, cases of rapid clinical decline have been seen when the drug is withdrawn [74]. In these situations, the decision to continue treatment with these drugs or not must be founded on both imaging criteria (progression in a single location and/or metastases appearing exclusively in the brain) as well as clinical criteria, considering the possibility of local treatment administration on the isolated areas of progression while maintaining TKI. Another important aspect in these patients is explaining the mechanism of resistance (change in the histological pattern with transformation to a small-cell variant, appearance of EGFR resistance mutations, such as T790 M, or association of new mutations), for which functional imaging can be tremendously helpful by enabling us to correctly guide biopsy toward areas of active tumor.

Immunotherapy has recently been incorporated into the treatment of advanced lung cancer by means of anti-PD-1 and anti-PD L1 antibodies (nivolumab, pembrolizumab, atezolizumab and others). The mechanism of action of these antibodies resides in the reactivation of the antitumor T-lymphocytes that are inhibited by the tumor itself. Several studies have shown their superiority in terms of response and tolerability versus conventional chemotherapy schedules [75]. The responses to these therapies often present unconventional patterns that are underestimated by morphological criteria, as they associate phenomena of delayed response and pseudoprogression, due to lymphocytic infiltration and inflammation. For this reason, new criteria of response to immunotherapy or irRC have been postulated [76]. As a comparison, in a recent study in patients with pembrolizumab treated melanoma, up to 12% of patients categorized on the basis of RECIST criteria were seen to be reclassified as responders by irRC and their overall survival was comparable to that of the cohort of responders based on irRC and RECIST [77].

After therapy, CT or ^18^F-FDG PET-CT can have important limitations in the differentiation between viable tumor and inflammation. However, other methods of functional imaging such as DWI has shown promising results to discern between fibrosis-inflammation and active tumor in this task, based on using the quantitative changes in ADC values and are potentially useful for early determination of response [70].

## Future perspectives for imaging techniques

**Fig. 8:**
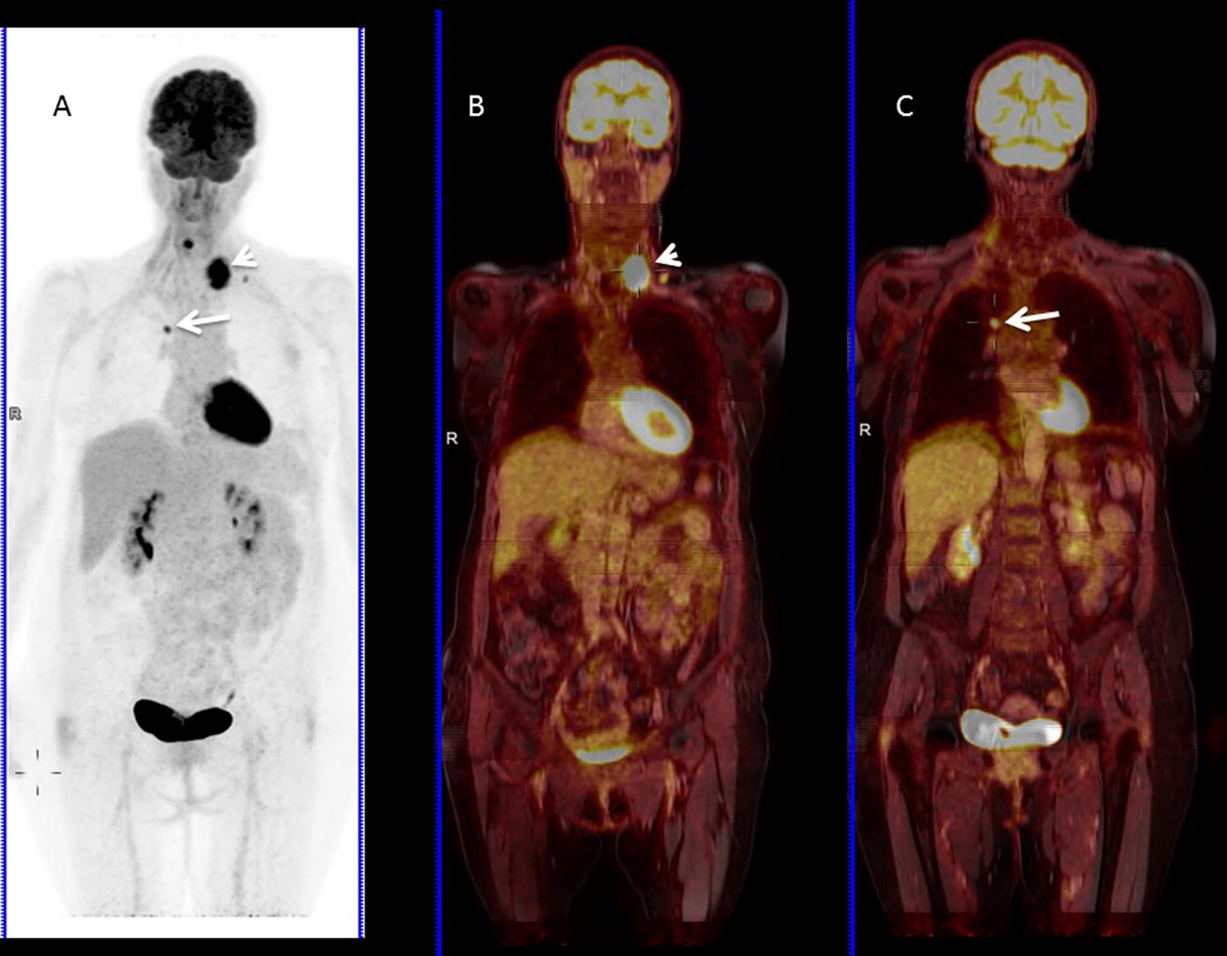
Follow-up of treated NSCLC with PET-MRI. **a** ^18^F-FDG PET and **b**, **c** fused PET and MRI images demonstrated shows a hypermetabolic small nodule in right upper lobe consistent with recurrent NSCLC (arrow) and secondary metastatic left superior mediastinal lymph node (arrowhead)

Hybrid ^18^F-FDG PET/MRI, integrating the conjoined acquisition of whole-body morphological MRI imaging combined with functional MRI sequences, such as DWI and dynamic-enhanced MRI imaging, and metabolic information from ^18^F-FDG PET, has been introduced in the clinical arena for the assessment of NSCLC [78] (Fig. 8).

The first advantage of PET/MRI is the lower dose of radiation in comparison to ^18^F-FDG PET/CT. Another advantage is the superior tissue characterization provided by MRI in comparison to CT, particularly for soft tissue tumors. Although, CT still continues to be the best imaging method to identify small pulmonary nodules, several studies have shown that pulmonary nodules that go undetected by ^18^F-FDG PET/MRI were benign, stable over time, or had disappeared on follow-up studies [79, 80]. ^18^F-FDG PET/MRI is the only truly simultaneous, multimodality technique, in which data acquisition is performed at the same time and, therefore, patient position is the same for both techniques, making it the perfect blend and highly valuable for small lesions. Furthermore, MRI has a wealth of sequences that are extremely useful, not only in diagnosis, but also for assessing prognosis and response. The combined use of DWI and/or perfusion sequences together with the advantages of ^18^F-FDG PET imaging is an important reason to believe in the expanded use of ^18^F-FDG PET/MRI in the near future for the assessment of NSCLC.

The main limitations are related to longer acquisition time and increased acquisition and maintenance costs in comparison to ^18^F-FDG PET/CT, and finally, ^18^F-FDG PET/CT still offers greater sensitivity in detecting pulmonary micronodules [81].

## Conclusions

1. Functional ^18F-^FDG PET/CT imaging plays a key role in staging NSCLC and is recommended by the leading clinical guidelines, particularly in surgical candidates or in cases in which radiotherapy is indicated.
2. Assessing response to chemotherapy and radiotherapy is the main challenge for the imaging techniques currently available in daily clinical practice. While the RECIST v1.1 criteria are the standard tool for evaluating response, morphological criteria are not capable of correctly assessing all clinical situations and that can lead to inadequate treatment decisions. Consequently, the most appropriate approach for response evaluation would be a combination of morphological, functional and molecular imaging techniques, especially when morphological alterations persist after treatment or in the case of the use of new treatment modalities. A progression of local metabolic findings on follow-up ^18^F-FDG PET scans or the detection of findings in distant locations suspicious of distant progression would have a significant impact on patient treatment and prognosis.
3. The incorporation of other complementary criteria, such as PERCIST or irRC, into medical practice and clinical trials would enhance anti-tumor response capacity and results.
4. Combined MRI protocols including structural sequences and DWI are a valid alternative to ^18^F-FDG PET/CT to characterize pulmonary nodules in some scenarios, as well as for NSCLC staging and treatment follow-up.
5. The use of perfusion CT and perfusion MRI are very promising for early monitoring of antiangiogenic drug treatment.
6. The incorporation of functional imaging techniques in clinical practice is considered as essential, although there is a need of prospective clinical trials to establish their appropriate usefulness.
7. The imaging specialist has a fundamental role to play in the initial diagnosis and staging of NSCLC, as well as in evaluating response. This specialist must make use of all the means already available to obtain relevant information from the different imaging studies. This would lead to maximizing already available resources.
8. Cooperation between clinicians and imaging specialists is imperative if advances in imaging techniques are to be optimally and timely applied to the management of each patient’s diagnostic and therapeutic circumstances. The use of functional and molecular imaging techniques can help in personalizing and monitoring NSCLC treatment. This collaboration calls for areas of interaction to be embedded in routine clinical activity. Actively engaging imaging specialists in Tumor Boards is one of the most efficacious tools in achieving these goals.

## Abbreviations

SEOM: Sociedad Española de Oncología Médica
SERAM: Sociedad Española de Radiología Médica
SEMNIM: Sociedad Española de Medicina Nuclear e Imagen Molecular
